# Combinational treatments of RNA interference and extracellular vesicles in the spinocerebellar ataxia

**DOI:** 10.3389/fnmol.2022.1043947

**Published:** 2022-10-13

**Authors:** Yingying Ding, Yong Zhang, Xuehong Liu

**Affiliations:** ^1^Department of Histology and Embryology, Medical College, Shaoxing University, Shaoxing, Zhejiang, China; ^2^Department of Clinical Medicine, Affiliated Hospital of Hangzhou Normal University, Hangzhou, China

**Keywords:** extracellular vesicle, RNA interference, miRNA, siRNA, shRNA, spinocerebellar ataxia, neurodegenerative disease, combinational treatment

## Abstract

Spinocerebellar ataxia (SCA) is an autosomal dominant neurodegenerative disease (ND) with a high mortality rate. Symptomatic treatment is the only clinically adopted treatment. However, it has poor effect and serious complications. Traditional diagnostic methods [such as magnetic resonance imaging (MRI)] have drawbacks. Presently, the superiority of RNA interference (RNAi) and extracellular vesicles (EVs) in improving SCA has attracted extensive attention. Both can serve as the potential biomarkers for the diagnosing and monitoring disease progression. Herein, we analyzed the basis and prospect of therapies for SCA. Meanwhile, we elaborated the development and application of miRNAs, siRNAs, shRNAs, and EVs in the diagnosis and treatment of SCA. We propose the combination of RNAi and EVs to avoid the adverse factors of their respective treatment and maximize the benefits of treatment through the technology of EVs loaded with RNA. Obviously, the combinational therapy of RNAi and EVs may more accurately diagnose and cure SCA.

## Introduction

Spinocerebellar ataxia (SCA) is a group of progressive neurodegenerative diseases (NDs) with autosomal dominant inheritance. They are divided into 48 subtypes according to the time sequence of discovery of the pathogenic gene loci. SCA has different clinical features, and ataxia is their core symptom (Chen H. Y. et al., 2021; Shin et al., 2021). The pathologically extended cytosine adenine guanine (CAG) repeat sequences encoded by poly-glutamine (polyQ) are their common pathogenesis, which leads to the misfolding and aggregation of translated protein and causes neuronal death (Shin et al., 2021). The prevalence of SCA is approximately 3/100,000 that varies in different subtypes and regions (Gómez et al., 2022). Currently, there are only several symptomatic treatments for SCA, which cannot prevent or delay the progression of SCA with severe complications. Patients usually die because brain stem lesions, pulmonary infection and respiratory failure within 10–30 years after diagnosis (Pérez Ortiz and Orr, 2018; Cendelin et al., 2022). Thyroid-stimulating hormone releasing hormone (TRH) can improve the symptoms (Shimizu et al., 2020). However, its complications such as headache and nausea occur in about 50% patients (Ghanekar et al., 2022). Aripiprazole improves dyskinesia but has significant side effects, such as anxiety, nausea, and dizziness (Jalles et al., 2022). The complex pathogenic mechanism and differences between subtypes, patients show the inadequacy of symptomatic treatment (Yap et al., 2022b).

Gene therapies, including RNA interference (RNAi), antisense oligonucleotides, and gene editing technology, are hoped to cure SCA by correcting the pathogenic genes (Vázquez-Mojena et al., 2021). RNAi is the most popular and effective way in preclinical trials mediated by microRNAs (miRNAs), small interfering RNAs (siRNAs), and short hair protein RNAs (shRNAs) (Costa, 2020). Repeat-targeting shRNAs specifically silence the expression of extended CAG repeats (Kotowska-Zimmer et al., 2020). Adeno-associated virus (AAV) loads with microRNA against ataxin (ATXN) 3 to restore the neuronal signaling and physiological functions in SCA3 mice (Nobre et al., 2022). Long-term and persistent RNAi reduces the cerebellar lesions and axonal demyelination in SCA3 mice by lowering the level of ATXN3 (Costa et al., 2020). However, RNAi has disadvantages such as carrier selection, poor stability *in vivo*, off-target effect and toxicity caused by excess effect (Vázquez-Mojena et al., 2021; Wang et al., 2022). Extracellular vesicles (EVs) as carriers have long plasma half-life, strong stability *in vivo* and can target the injury site (Sun et al., 2010; Huang et al., 2020). miRNA is an important component of EVs, which can treat SCA. Mesenchymal stem cell (MSC)-derived exosomes can alleviate the damage to Purkinje fibers and myelin sheath, motor function and neuroinflammation in SCA3 mice (You et al., 2020; Tao et al., 2021). The miRNA in EVs can be biomarkers of SCA3, revealing the subtypes and progression of SCA3 (Hou et al., 2019). Obviously, the combination of RNAi and EVs can avoid the adverse factors of RNAi or EVs treatment alone to maximize the benefits of treatment. The combinational therapy of RNAi and EVs is a brand new idea. It may more accurately diagnose and cure SCA, which still needs our in-depth research.

## RNA interference

RNA interference is a process, which is mediated by double-stranded RNA (dsRNA) and leads to the silence of target genes (mRNA) containing complementary sequences and inhibition of related protein production (Kara et al., 2022). dsRNAs in the process include miRNAs, siRNAs and shRNAs (Costa, 2020). The pathogenesis of NDs remains unknown. However, their pathological features are the deposition of misfolded protein such as amyloid-β, alpha-synuclein, tau and prion proteins. RNAi can completely eliminate or alter the synthesis of target protein (Khan et al., 2022). RNAi can treat SCA by silencing the specific genes and assisting in the diagnosis and condition monitoring of SCA.

### microRNAs

microRNAs are single-stranded non-coding RNAs, about 19–25 nucleotides in length. They are RNAi silencing trigger that binds to a specific region of 3′ untranslated region of messenger RNA, silences the target gene by altering RNA stability to inhibit the corresponding protein synthesis (Wei et al., 2021). miRNAs are divided into artificial synthesis miRNAs and natural synthesis miRNAs. Artificial miRNAs can target disease genes (Saad et al., 2021). They are important for brain development and homeostasis (Penning et al., 2021). The imbalance of miRNAs is closely related to the pathogenesis of NDs, which may be the biomarkers. The expressions of Hsa-miR-204-3p and hsa-miR-873-3p are significantly increased, and the expression of hsa-miR-6840-5p is decreased in the patients of progressive supranuclear palsy (Nonaka et al., 2022). The expressions of miR-132-5p, miR-138-5p, and miR-129-5p are reduced in the brain of Alzheimer’s disease (AD) patients (Dobricic et al., 2022). MIR-144 expression is significantly increased in Multiple Sclerosis (MS) patients (Roshani et al., 2021). miR-181 can be a potential biomarker for the prognosis of amyotrophic lateral sclerosis (ALS). Patients with low miR-181 content have a longer survival time (Magen et al., 2021).

microRNAs are important for the treatment of NDs. The increased serum miR-485-3p can be a diagnostic marker for AD. Knocking out miR-485-3p reduces neuro-inflammation and improves neuronal viability, which is conducive to protect neurons in AD patients (Yu et al., 2021). Injection of AAV encoding miRNA targets to the superoxide dismutase 1 and immunosuppressive agents to improve sensory dysfunction and delay disease progression in an ALS patient (Mueller et al., 2020). miRNA inhibits the effect of stress on visual transduction and reduces the retinal inflammation to treat the age-related macular degeneration (Chu-Tan et al., 2021).

microRNAs are potential in the diagnosis, treatment and monitoring of SCA. Three miRNAs interact with ATXN3-3′UTR (miR-9, mir-181a, and mir-494) that are overexpressed in SCA3 patients (Carmona et al., 2017). The injection of AAV expressing miR760 reduces ATXN1 levels *in vivo* and improves motor disharmony in the cerebellum of SCA1 mice (Nitschke et al., 2020). miR-32 and miR-181c target the 3′UTR of ATXN3 and inhibit the expression of ATXN3 to reduce the polyQ-mediated cytotoxicity (Krauss et al., 2019). AAV5-loaded miRNA ATXN3 specifically decreases the ATXN3 mRNA and protein in SCA3 mice and cell models (Martier et al., 2019). The lowest effective dose, the maximum tolerated dose and the toxicity threshold that can improve SCA1 to guide the clinical application of RNAi in the treatment of SCA1 (Keiser et al., 2016).

### Small interfering RNAs (siRNAs)

Small interfering RNAs are short dsRNAs of 20 to 25 base pairs produced by RNAse III family cutting long dsRNAs. They silence the post-transcription genes by interfering with the expression of specific genes through complementary nucleotides (Jiang et al., 2021; Akhilesh et al., 2022). In mammals, siRNAs only come from synthetic sources (Wang et al., 2021).

Small interfering RNAs participate in various biological mechanisms, including the proliferation, growth and differentiation of cells, as well as the arrangement of heterochromatin in nucleus (Mishra et al., 2022). SiRNA-mediated glycogen synthase kinase (GSK)3β silencing improves the cognitive function and relieves the progression of AD by eliminating the deposition of neurofibrils and amyloid plaques (Gupta et al., 2022). SiRNA restores the normal expression of schwann cells in Charcot-Marie-tooth disease type 1a mice, electrophysiological activity of motor nerves and muscular function. However, long-term treatment produces off-target and toxic side effects (Boutary et al., 2021). Anti-Huntington (HTT) siRNAs effectively reduce the expression of HTT gene and slow the disease’s progression in mice with Huntington’s disease (HD) (Sava et al., 2020).

CAG-siRNA specifically silences the alleles of estrogen receptor with CAG repeats in fibroblasts, inhibits the generation of polyQ protein in the central nervous system to treat the SCA, spinal cord and medullary atrophy, HD, dentatered-globus pallidus atrophy, etc., (Hirunagi et al., 2021). Intravenous siRNAs reduce the Axin-3 expression, alleviate the dyskinesia and improve the striatal and cerebellar lesions in SCA3 mice (Conceição et al., 2016).

### Short hairpin RNAs (shRNAs)

Short hairpin RNA consists of two complementary 19–22 bp RNA sequences linked by a short loop consisting of 4–11 nt. It is similar to the hairpin in naturally synthesize miRNA, so it is called the short hairpin RNA (Moore et al., 2010). Although shRNAs are exogenous RNAs (Sheng et al., 2020), they can be assimilated into endogenous RNA pathways with greater efficiency than siRNAs (Gorabi et al., 2020).

Short hairpin RNAs inhibit the expressions of SNCA and endogenous α-synuclein to protect nerves and reduce dyskinesia in the substantia nigra of Parkinson’s disease (PD) rats (Nakamori et al., 2019). shRNAs convert the midbrain astrocytes into dopaminergic neurons to improve the motor dysfunction in PD (Qian et al., 2020). shRNAs target the glutamate N-methyl-d-aspartate receptor subunit (GluN) 3A inhibition to prevent the loss of dendritic spines and improve the locomotor performance in HD mice (Marco et al., 2018). shRNAs carry out spindle pole body component (SPC) 25 to pass through the blood-brain barrier (BBB), reduce the proliferation of microglia, and slow the disease progression in AD mice (Cui et al., 2021). For SCA3 and SCA7, shRNAs reduce the Axin-3 in mutant cells by 60%, and the expressions and aggregations of toxic mutant ataxin-7 proteins. The normal proteins are unchanged and stored in the non-aggregated diffuse cells (Scholefield et al., 2009; Kotowska-Zimmer et al., 2020). shRNAs restore the cerebellar morphology of SCA1 mice and improve the motor coordination of Purkinje cells (Xia et al., 2004).

The currently RNAi drugs only include N-acetylgalactosamine-siRNA conjugates (givosiran, inclisiran, and lumasiran), which targets to hepatocyte and siRNA drugs (vutrisiran and onpattro) that can treat the familial amyloid polyneuropathy (Kulkarni et al., 2021; Holm et al., 2022). Although RNAi shows the different ways of gene silencing in each ND, it seems to be a general treatment for them. However, it suffers from endosome escape, off-target effect, unclear targeting specificity, and short half-life (the half-life is only a few minutes due to rapid degradation by nucleases) (Aigner, 2019; Setten et al., 2019). Conventional RNAi vectors have shortcomings. Adenoviral vectors themselves can be toxic (Keiser et al., 2021). Liposomes have a short half-life due to the hydrolysis and oxidation of phospholipids and are vulnerable to temperature. Composite nanoparticle are cytotoxic which may destroy the genetic material of a body (Hernández-Soto and Chacón-Cerdas, 2021). The aforementioned disadvantages of RNAi make it difficult to treat SCA alone.

## Extracellular vesicles

Extracellular vesicles have target specificity, long plasma half-life and wide distribution, and can cross the BBB. Their contents such as miRNAs can assist in the diagnosis and prognosis of NDs. Neurotrophic factors (NTFs), heat shock proteins, vascular endothelial growth factors and other factors can restore neuronal activity and maintain protein homeostasis.

### The general position of extracellular vesicles

Extracellular vesicles are nano-sized vesicles released by various cells and surrounded by phospholipid membranes (Ye et al., 2022). EVs contain abundant DNA, proteins, lipids, mRNA and small non-coding RNA, including miRNAs (account for half of the content of EVs), which are not affected by extracellular proteases and nucleases (Cuomo-Haymour et al., 2022; Zhu et al., 2022). According to the mechanism and diameter of the production, they are divided into exosomes (50–150 nm), microbubbles (100–1,000 nm) and apoptotic bodies (100–5,000 nm). Their functions are related to the originating cells (Mathieu et al., 2019).

As a novel carrier, EVs have high biocompatibility to avoid the adverse immune reactions, targeting specificity, low toxicity, long plasma half-life, source diversity, and can be easy to cross the BBB to overcome the shortcomings of conventional carriers and RNAi (Zhang et al., 2019; O’Brien et al., 2020; Herrmann et al., 2021).

Extracellular vesicles are important in the treatment of NDs (Table 1). Stem cell-derived EVs have neuroprotective and immunomodulatory effects (Niu et al., 2020; Garcia-Contreras and Thakor, 2021). EVs derive from MSCs and anti-inflammatory immune cells that can reduce the inflammatory damage and cell stress (Thome et al., 2022). The exosomes of human amniotic fluid MSCs can treat the inflammation-related neurological diseases by reducing inflammation caused by microglia and improving neurotoxicity (Zavatti et al., 2022). EV-mediated the delivery of DnaJ Homolog Subfamily B Member 6 (DNAJB6) molecular chaperone inhibits the aggregation of polyQ and HTT proteins to delay the onset of HD (Joshi et al., 2021). EVs derive from neural stem cells that target the functional substances to protect damaged neurons by resisting oxidation, apoptosis and inflammation (Lee et al., 2022). Most of the data on the treatment of EVs are concentrated in preclinical trials, but many clinical trials have proved the potential of EVs in diagnosis and monitoring.

**TABLE 1:** Clinical treatment of EVs in the neurodegenerative diseases.

Sources of EVs	Diseases	Species	Treatment options	Outcomes	References
Human bone marrow-derived mesenchymal stem cell (MSC)	Status epilepticus	Mouse	EVs derived from human bone marrow-derived MSCs are administered intranasally (15 μg, ∼7.5 × 109)	Inflammatory response, abnormal neurogenesis, memory and cognitive deficits are significantly inhibited.	Long et al., 2017
Bone-marrow mesenchymal stem cells (BMSCs)	Alzheimer’s disease (AD)	Mouse	BMSC-exos are administered intraventricular (0.5 μg per day)	Inflammatory response, glial cell over-activation are significantly improve and the expression of BDNF is significantly increased.	Liu S. et al., 2022
GDNF transfected macrophages	Parkinson disease (PD)	Mouse	PD mice are intranasally dosed with EV-GDNF three times per week (3 × 109 particles/10 μl/mouse)	The activity of mice significantly improved, neurons increased, inflammatory response decreased, and no significant toxic reactions are observed.	Zhao et al., 2022a
Blood from healthy volunteers	PD	Mouse	PD mice are given exosomes via tail vein (0.2 ml per mouse, four injections)	Motor coordination is restored with increased dopaminergic neuron production in the substantia nigra and striatum, and oxidative stress, neuroinflammation, and apoptosis are decreased.	Sun et al., 2020
Astrocyte	Traumatic brain injury (TBI)	Rat	Exosomes are injected into the lateral ventricles of each cerebral hemisphere of the rat (2 μl of 2,400 × -enriched exosomes)	Oxidative stress is reduced and the damaged mitochondria and neurons are restored.	Chen et al., 2020
Human MSC	TBI	Swine	Exosomes are injected through the left external jugular vein slowly (1 ml/min, 1 × 10 exosome particles in 5 ml lactated Ringer’s)	Exosomes treatment significantly reduces brain swelling and lesion size, reduces brain biomarkers, and improves the integrity of the blood-brain barrier.	Williams et al., 2020
Serum from young mice	Huntington’s disease (HD)	Cell	Young serum-exosomes are administered to the HD cell model. (200 μg/ml for 3 days)	The decrease of mHtt agglutinin and apoptosis signal makes more cells survive, less apoptosis and mitochondrial dysfunction recover.	Lee et al., 2021
Adipose-derived stem cells (ASCs)	Amyotrophic lateral sclerosis (ALS)	Mouse	Exosomes (6~8 × 108/ml) are administered intravenously and intranasally to the mice. (The iv dose was 100 μl or 10 μl).	Movement is improved with improved lumbar motor neurons, neuromuscular junctions and muscle function, and decreases glial cell activity.	Bonafede et al., 2020
ASCs from mice	ALS	Mouse	NSC-34 cells are inoculated in medium with or without 0.2 μg/ml exosomes.	Decreasing pro-apoptotic proteins, increasing anti-apoptotic proteins and cell viability provide neuroprotective effects	Bonafede et al., 2019

Extracellular vesicles can assist in the diagnosis and monitoring of NDs (Table 2). The α-globin, β-globin, δ-globin, α-1-antichymotrypsin, beta-2-glycoprotein 1 and complement component C9 are increased, and apolipoprotein C-III is decreased in the neuron-derived exosomes of AD patients (Arioz et al., 2021; Soares Martins et al., 2022). The number of α-syn-carrying EVs in cerebrospinal fluid (CSF) and plasma of PD patients are significantly increased, which is positively correlated with the severity (Agliardi et al., 2021; Hong et al., 2021). Concentrations of syntaxin-1A (STX-1A) and vesicle associated membrane protein 2 (VAMP-2) are significantly reduced in PD (Agliardi et al., 2021). The concentration of toll-like receptor (TLR) 3 is decreased and TLR4 is increased in EVs from MS patients (Bhargava et al., 2019). The increased expressions of Junctional Adhesion Molecule A (JAM-A), TNF-R2, and Chitinase 1 lead to increase platelet activation, angiogenesis, motor neuron destruction and microglia activation in the EVs of ALS patients (Czubak-Prowizor et al., 2022; Sjoqvist and Otake, 2022). In the clinical trial of exenatide for PD, neuron-derived EVs, as biomarkers, have potential in examining the molecular mechanisms of patients’ motor function changes (Athauda et al., 2019).

**TABLE 2:** EVs as the biological markers of neurodegenerative diseases.

Diseases	Species	Source of EVs	Contents of EVs	Significance of changes	References
AD	Human	Neuron-derived plasma EVs	High brain β-amyloid (Aβ)	It is suggested that cognitive function are impaired, entorhinal atrophy and amyloid deposition in cortex.	Li T. R. et al., 2022
AD	Human	Central nervous system-derived plasma EVs	NMDAR2A	It may be related to impaired synaptic function in the brain.	Tian et al., 2022
Chronic Traumatic Encephalopathy (CTE)	Human	CTE brain tissues	t-tau, p-tau, SNAP-25, PLXNA4	It is related to further development of the disease.	Muraoka et al., 2021b
CTE	Human	Plasma	COL6A3, RELN and COL6A1	They, respectively, indicate impaired sciatic nerve, increased tau phosphorylation, and rarefaction of white matter.	Muraoka et al., 2021a
PD	Human	Neuron-derived plasma EVs	α-synuclein, Phosphorylated Tau (T181)	They indicate that PD patients have cognitive impairment, which can predict the cognitive prognosis.	Blommer et al., 2022
PD	Human	Plasma	Neurofilament light chain (NfL)	NfL may be related to the degree of dyskinesia in PD patients, especially in terms of akinetic rigidity.	Chung et al., 2020
Huntington’s disease (HD)	Human and Mouse	Cerebrospinal fluid (CSF)	SG-nucleating Ras GTPase-activating protein-binding protein 1 (G3BP1)	It is related to the pathogenesis of HD. The increased production in cortex and hippocampal pyramidal neurons may be related to the memory impairment of HD patients.	Sanchez et al., 2021
ALS	Human	CSF and plasma	neurofilament light chain (NfL), TAR DNA-binding protein 43 (TDP-43), and total	All of them can be used as sensitive biomarkers for the diagnosis of ALS. NFL can predict disease progression	Kasai et al., 2019
Sporadic Creutzfeldt-Jakob disease (sCJD)	Human	CSF	tau (t-tau) 14-3-3β,14-3-3γ NfL, neuron-specific enolase, RT-QuIC, S100B, t-tau	RT-QuIC is the most accurate biomarkers. RT-QuIC and NfL are the most sensitive diagnostic marker.	Rübsamen et al., 2022
Epilepsy	Human and Mouse	Serum	coagulation factor IX (F9) and thrombospondin-1 (TSP-1)	F9 may be associated with excitatory toxicity, apoptosis, and coagulation abnormalities. TSP-1 may be related to abnormally high excitability.	Lin et al., 2020

### The use of extracellular vesicles in spinocerebellar ataxia

**FIGURE 1:**
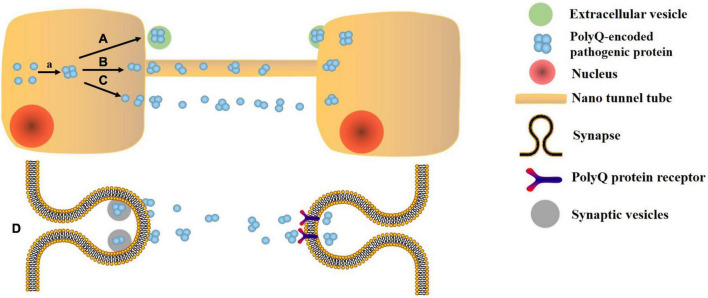
Transmission mechanism of polyQ protein. a. Error folding and aggregation of polyQ encoded protein. A. Extracellular vesicle pathway. B. Nano tunnel tube approach: the polyQ protein is exchanged by a tunnel-like membrane-derived structure that connects two cells. C. Soluble oligomer: due to its high solubility and small size, PolyQ protein may be able to escape from cells and diffuse to other cells. D. Synaptic connection: the polyQ protein is transmitted between neurons with high transmission efficiency.

Extracellular vesicles, as an important communication tool, can transmit the pathogenic gene, misfold, and aggregate proteins between cells, thus participating in the pathophysiology of NDs (Surgucheva et al., 2012). Other propagation mechanisms include soluble oligomers, synaptic connections, and nano tunnel tube (Takeuchi and Nagai, 2022; Figure 1). As EVs contain miRNAs, proteins and other components, their changes can reflect the condition of SCA (Cuomo-Haymour et al., 2022). There are few studies in the biomarkers of EVs in SCA compared to AD, PD, and ALS. Only one study of miRNA in the EVs is described below. None of the studies about protein biomarkers has demonstrated that they are in EVs. Both neurofilament medium (NFL) and myelin basic protein levels decrease in the cerebellar cortex of SCA3 patients, indicating axonal damage and myelin formation disorders (Paul et al., 2018). Increased levels of staufen 1 are associated with the abnormal metabolism of RNA due to ATXN2 mutations in the brain tissue of SCA2 patients (Yang et al., 2021). The traditional diagnostic methods for SCA include three aspects: (1) MRI can only show the pathological changes of extensive atrophy of the brain and thinning of the cortex, which lacks the functional changes corresponding to the pathological changes. Meanwhile, MRI is very expensive (Yap et al., 2022a). (2) The diagnosis of symptomatology is easily affected by subjectivity. (3) Genetic examination: The gene loci of mutations in different subtypes are different. For example, SCA1, SCA2, SCA3, SCA6, SCA7, SCA12, and SCA19/22 are, respectively, related to the CAG repeated amplification in ATXN1, ATXN2, ATXN3, CACNA1A, ATX7, PPP2R2B, and potassium voltage gated channel, Shal-related family, member 3 (KCND3) genes on different chromosomes (Li M. et al., 2022; Lian et al., 2022).

Extracellular vesicles can transmit NTFs, heat shock proteins, and others to promote the damaged neurons and maintain the stability and activity of proteins (Backe et al., 2022; Harrell et al., 2022; Van den Broek et al., 2022). The proteins encoded by polyQ are the key to the pathogenesis of SCA, and Heat shock protein (HSP) 70 can promote the synthesis of correctly folded proteins and the degradation of misfolded proteins, prevent the aggregation of abnormal proteins and disaggregate the aggregation of abnormal proteins (Johnson et al., 2020; Serlidaki et al., 2020). MSCs-derived exosomes reduce the loss of purkinje cells and cerebellar myelin sheaths, alleviate neuroinflammation and improve the locomotor function in SCA3 mice [19] (You et al., 2020). MSC-conditioned medium (simulates EVs) significantly reduces the motor disharmony and neuronal degeneration in SCA1 mice, due to the inclusion of hepatocyte growth factor, fibroblast growth factor-2, insulin-like growth factor-1 and vascular endothelial growth factor (VEGF) (Suto et al., 2016). Studies have shown that VEGF can reduce ataxia and dendritic defects and even reverse cerebellar lesions in SCA1 (Hu et al., 2019).

Extracellular vesicles also face some challenges, which have been nowly solved. Endogenous EVs competing for binding sites and the clearance of macrophages can be solved by specifically modifying the surface of EVs (Zhang et al., 2022). EVs from the different sources have the different effects. Novel micro-nano technology tool can sensitively separate the different subsets of EVs (Chiang and Chen, 2019; Wang et al., 2020). Ultra-centrifugation, ultrafiltration, precipitation and size exclusion chromatography improve the separation and purification of EVs (Li M. et al., 2020). Membrane extrusion technology combine the surface composition of EVs with lipid materials to achieve mass production of EVs (Jhan et al., 2020).

## The combinational therapeutic effect of RNA interference and extracellular vesicles in spinocerebellar ataxia

Extracellular vesicles not only can treat SCA, but also can be carriers of RNAi, which can make up for the deficiencies of RNAi. Treating SCA with the combination of EVs and RNAi is a promising direction. The miRNAs and proteins contained in EVs will undergo corresponding significant changes, making them biomarkers to assist the diagnosis and monitoring of SCA.

### A novel RNA interference delivery carrier – Extracellular vesicles

Several cells can self-secrete miRNAs through paracrine and transport them to the receptor cells, and then change the expression of target genes, such as macrophages, microglia, oligodendrocytes, astrocytes, schwann cells and so on (Loch-Neckel et al., 2022; Zhu et al., 2022). Astrocyte-derived exosomes carrying miR-17-5p reduce oxidative stress, inflammatory, cerebral infarction and neurological disorders in hypoxic-ischemic brain injured rats (Du et al., 2021). Microglia secretes the exosomes containing miR-190b to inhibit neuronal apoptosis by targeting autophagy-related protein 7 to regulate autophagy (Pei et al., 2020). M2 microglia secretes the exosomes containing miR-124 to target ubiquitin-specific protease 14 to promote neuronal survival and mitigate ischemic brain injury (Song et al., 2019). Fibroblasts secrete the exosomes containing miR-673-5p to stimulate myelin gene expression by activating the tuberous sclerosis complex 2/mTOR complex 1/sterol-regulatory element binding protein 2 axis in Schwann cells and peripheral myelin formation (Zhao et al., 2022b). It is infeasible to implant them directly to patients. On the one hand, they may contain pathogenic substances. The transmission of astrocyte-derived EVs carrying casein kinase 1 increases the formation of Aβ Aand promotes inflammatory response by targeting the related neurons, which is the pathogenesis of AD (Li Z. et al., 2020). EVs can spread α-synuclein in the brain and accelerate the progress of PD [126] (Longoni et al., 2019). On the other hand, as there are 48 subtypes of SCA having the different genetic changes which require the different types of RNAi (Chen H. Y. et al., 2021; Shin et al., 2021). The composition and function of EVs are related to its secretory cells and their environments. The MSC-derived EVs in an inflammatory environment reduce inflammatory response more than the normal MSC-derived EVs (Mathieu et al., 2019; Liu Y. et al., 2022). Hypoxia-treated exosomes improve the inflammatory responses and synaptic functions by increasing the production of miR-21 to improve the cognitive and memory disorders (Cui et al., 2018). Therefore, we should select the metrocyte that can alleviate nerve damage and movement disorder, and appropriately stimulate the metrocyte to maximize the therapeutic value of EVs derived from it.

**FIGURE 2:**
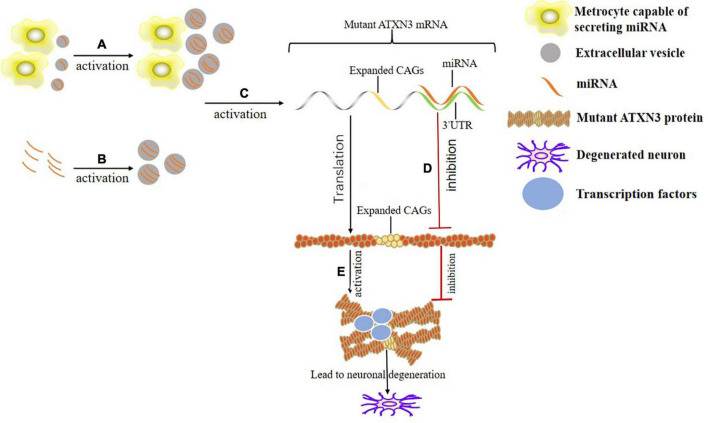
Mechanism of the binding of EVs and miRNA in the treatment of SCA3. **(A)** Appropriately stimulate the specific metrocyte (such as hypoxia treatment) to maximize the therapeutic value of EVs derived from it. **(B)** Load the desired miRNA into the isolated and purified EVs through electroporation, incubation or ultrasound, etc. **(C)** EVs with specific miRNA acts on corresponding miRNA sites (there are 1,137 known sites presently) in the 3’UTR of mutant ATXN3 mRNA. **(D)** The mutated ATXN3 mRNA that binds to the miRNA in the 3’UTR region is degraded or its translation is inhibited. **(E)** Misfolding and aggregation of the mutated ATXN3 protein occur. Transcription factors are adsorbed by protein aggregates, interfering with the expression and biogenesis of normal miRNA. Eventually the neurons degenerate.

The desired RNAi can be loaded into the isolated and purified EVs. Electroporation is the most common and optimal technology. Each EV is probably loaded with 3,000 miRNA molecules (Batrakova and Kim, 2015; Pomatto et al., 2022). Incubation is a simple method while maintaining the integrity and stability of the EVs. It is achieved by incubating EVs with miRNA in Roswell Park Memorial Institute (RPMI) medium at 37°C for 1 h (Tapparo et al., 2021). Ultrasound damages the integrity of EVs membranes, in which drugs, protein and nanoparticles are loaded, and then membranes are recovered by incubation (Li Y. J. et al., 2020). Under the cross substitution of heat shock and freezing, CaCl_2_ mediates the loading of miRNA into the exosomes without losing activity and with high efficiency (Rezaei et al., 2021). Although the method of encapsulating RNAi in EVs has not been tested in SCA, many studies have proved the feasibility in NDs, autoimmune diseases, tumors, cardiovascular diseases, etc. The EVs-derived from human neural stem cells attenuate the neuroinflammation and ROS-induced apoptosis, and promote the survival of dopaminergic neurons in PD, which may be related to the specific miRNA-embedded in EVs (Lee et al., 2022). Increasing the paracrine miR-146a of cortical astrocytes eliminate their phenotypic abnormalities through pre-miR-146a or dipeptidyl vinyl sulfone and play a neuro-glial repair role in ALS (Barbosa et al., 2021). Exosomes-loaded with miRNA-22 improve AD by inhibiting the pyroptosis and neuroinflammation (Zhai et al., 2021). The miRNA-encapsulated in exosomes control the target genes (nuclear factor kappa-B (NF-κB), signal transducer and activator of transcription (STAT) 1, and phosphatidylinositol 3 kinase/protein kinase B (PI3K/Akt), etc.) in the regulatory signaling pathway in immune response to treat the autoimmune diseases (Matsuzaka and Yashiro, 2022). Exosomes-loaded with miR-145-5p reduce the growth of pancreatic ductal adenocarcinoma (PDAC) by inhibiting the proliferation and invasion of PDAC cells (Ding et al., 2019). Exosomes carrying mir-6785-5P inhibit the angiogenesis and metastasis by inhibiting the inhibin subunit beta A in gastric cancer (Chen Z. et al., 2021). Cardiac progenitor cell-derived exosomes containing miR-146, miR-210, and miR-132 reduce the infarct size and improve the cardiac functions in infarcted hearts (Cervio et al., 2015; Figure 2).

### A novel spinocerebellar ataxia biomarker – RNA interference in extracellular vesicles

The onset and progression are vary significantly among SCA patients. Biomarkers can sensitively monitor the SCA progression that is needed (Borgonio-Cuadra et al., 2019). Conventional diagnostic methods for SCA have shortcomings and cannot accurately monitor the disease progression. Existing measurements of protein biomarkers such as NfL in CSF and blood fail to locate the site of injury (Yap et al., 2022a). Ataxin-3 produced by polyQ amplification in plasma is highly stable only for 1 year (Hübener-Schmid et al., 2021). The protein biomarkers in EVs may specifically concentrate at the injured site and remain stable for longer time unaffected by proteolytic enzymes.

The miRNA in exosomes directly reflect the physiological conditions of brain, which are highly expressed in brain and body fluids, such as serum, plasma and CSF (Mori et al., 2019). Furthermore, a DNA-assembled advanced plasmonic architecture-based plasmonic biosensor is sensitive and accurate enough to detect miRNA in exosomes for clinical applications (Song et al., 2022). The miRNAs in the SCA patients undergo the specific changes with low stability. Loading miRNA in EVs may protect them from nuclease-rich environments (Borgonio-Cuadra et al., 2019). The miR-7014 in EVs of SCA3 patients is downregulated in plasma and upregulated in CSF (Hou et al., 2019). Although there is only one case of RNAi in EVs as SCA biomarkers, its feasibility has been confirmed in cancer, autoimmune diseases, kidney diseases, and NDs. miR-320a and miR-4433b-5p in EVs can differentiate breast cancer from the control population (Carvalho et al., 2022). Blood-derived EVs show that the level of miRNA-146a is reduced in the systemic lupus erythematosus patients, as oppose to urine-derived EVs (Zhou et al., 2022). Renal function is negatively correlated with the levels of miRNA-12136 and miRNA-483-5p (Sun et al., 2022). Expression of miR-16-5p, -331-3p, -409-3p, and -454-3p increase 1.5-fold in the CSF-derived EVs in AD patients (Sandau et al., 2022). The combination of miR-125b and -361 in exosomes can distinguish AD patients from healthy control with an accuracy rate of 99.52% (Song et al., 2022). MS patients show the upregulation of miR-21-5p and the downregulation of miR-6735-3p, miR-6833-5p, and miR-510-3p in the EVs (Cuomo-Haymour et al., 2022). Hsa-miR-374a-5, -374b-5p,-199a-3p, -28-5p, -22-5p, and -151a-5p are potential biomarkers for the diagnosis of PD at different stages (He et al., 2021). Therefore, RNAi in EVs can assist in the diagnosis and monitoring of NDs.

There are still some limitations in the research of RNAi biomarkers in EVs. Most studies on biomarkers are conducted in SCA3 because it is the most common hypotype. Most RNAi studies have focused on miRNA because it accounts for half of the EVs content. Since SCA is a rare disease, the sample size of cases is insufficient, leading to the possibility of biased results. EVs and RNAi are affected by many factors *in vitro* and *in vivo*, so currently they can only serve as reference indicators for the diagnosis and monitoring of SCA.

## Conclusion and future prospect

At present, symptomatic treatment is the only clinical treatment for SCA that has many side effects and limited efficacy. Gene therapy is the most popular potential treatment for SCA, and RNAi is the most effective among it. Many studies have shown that EVs and RNAi work well in SCA cells, animal models, and patients. In particular, the combination of them can make up for their deficiencies. The therapeutic effect, target specificity, stability and plasma half-life of EVs containing RNAi secreted by cells or EVs loaded with specific RNAi are significantly improved. They also have potential in the diagnosis and condition monitoring of SCA. Changes of RNAi in patients’ EVs may reflect disease progression. However, there are few studies on the combination treatment, diagnosis and monitoring for SCA. In addition, most of them are animal and cell experiments, which do not eliminate the influence of sample size, gender, age, and race of the experimental subjects. Most studies have focused on SCA3 and miRNA while ignoring other subtypes of SCA and RNAi. Therefore, the combination of RNAi and EVs is a new therapeutic way for SCA, further in-depth clinical studies on large-scale multiple subtypes of RNAi and EVs are needed.

## Author contributions

XL and YD designed the study. YD, YZ, and XL prepared the first draft of the manuscript and revised the manuscript. All authors agreed to publish this article and approved the final manuscript.
